# The impact of sex on blood pressure and anthropometry trajectories from early adulthood in a Nigerian population: insights into women’s cardiovascular disease risk across the lifespan

**DOI:** 10.1186/s12905-022-01888-7

**Published:** 2022-07-22

**Authors:** Oluseyi Adegoke, Oluwadamilola O. Ojo, Obianuju B. Ozoh, Ayesha O. Akinkugbe, Ifedayo A. Odeniyi, Babawale T. Bello, Osigwe P. Agabi, Njideka U. Okubadejo

**Affiliations:** 1 Department of Medicine, Faculty of Clinical Sciences, College of Medicine, University of Lagos, Idi-Araba, PMB 12003 Lagos State Nigeria; 2 Lagos University Teaching Hospital, Idi-Araba, Lagos State Nigeria

**Keywords:** Blood pressure, Trajectory, Anthropometry, Age, Female sex, Black Africans

## Abstract

**Background:**

Sex disparities in blood pressure and anthropometry may account for differences in cardiovascular (CV) risk burden with advancing age; modulated by ethnic variability. We explored trajectories of blood pressures (BPs) and anthropometric indices with age on the basis of sex in an urban Nigerian population.

**Methods:**

We conducted a secondary analysis on data from 5135 participants (aged 16–92 years; 2671(52%) females) from our population-based cross-sectional study of BP profiles. We utilized the WHO STEPS and standardized methods for documenting BPs, body mass index (BMI) and waist circumference (WC). Data was analyzed using Analysis of variance (ANOVA), Spearman correlation analysis and mean difference in variables (with 95% confidence interval). We explored the influence of age and sex on BP profiles and specific anthropometric indices using generalized regression analysis.

**Results:**

In those aged 15–44 years, males had significantly higher systolic BP (SBP) and pulse pressure (PP). However, mean SBP and PP rose more steeply in females from 25 to 34 years, intersected with that of males from 45 to 54 years and remained consistently higher. Difference in mean BPs (95% Confidence Interval) (comparing < and > 45 years) was higher in females compared to males for SBP (17.4 (15.8 to 19.0) v. 9.2 (7.7 to 10.7), DBP (9.0 (7.9 to 10.1) v. 7.8 (6.7 to 8.9)), and PP (8.4 (7.3 to 9.5) v. 1.4 (0.3 to 2.5)). Females had significantly higher BMI and WC across all age groups (p < 0.001). Age more significantly correlated with BPs, BMI and WC in females. Interaction models revealed that SBP was significantly predicted by age category in females from (15–54 years), while DBP was only significantly predicted by age in the 15–34-year category (*p* < 0.01). BMI and WC were significantly predicted by age only in the 25–34-year category in females, (*p* < 0.01).

**Conclusions:**

Our population demonstrates sex disparity in trajectories of SBP, PP, BMI and WC with age; with steeper rise in females. There is a need to focus on CV risk reduction in females, starting before, or during early adulthood.

**Supplementary Information:**

The online version contains supplementary material available at 10.1186/s12905-022-01888-7.

## Introduction

Blood pressure and body adiposity are broadly recognized determinants of cardiovascular disease (CVD) risk [1, 2]. Reported variations in cardiovascular outcomes in men versus women have previously been attributed to sex-specific disparities in body adiposity and blood pressure trajectories, and are exemplified by differences in incidence, prevalence, disease patterns and profiles, and mortality [3–6]. As examples, high SBP has a higher global attributable disability-adjusted life years (DALYs) for females compared to males and pulse pressure (which is also associated with subclinical CVD) is modulated by sex [8]. Beyond the physiological and hormonal import of sex differences on vascular function, intersecting social factors such as disparate health-seeking behavior, access to healthcare, environmental and lifestyle dissimilarities may be contributory [9–11].

Any sex disparity in CV risk and outcomes deserves recognition due to the implications for risk management, surveillance and development of sex-specific guidelines guided by evidence [3–5, 12–14]. Although sex specific blood pressure trajectories and the implied CVD related risks have been reported, these are subject to wide racial, ethnic, geographic and cultural differences which are critical in planning interventions to reduce CVD-related adverse outcomes [8, 15–17]. The steeper rise in blood pressure reported to occur among women has shown variations in age of onset across populations [4, 8, 18, 19]. Furthermore, although measures of body adiposity (BMI and WC) have been independently associated with CVD risk; age, sex and ethnic variability have also been reported with these measures [20–24].

There is increasing incidence and prevalence of CV diseases in sub-Saharan Africa (SSA) with lower age of onset, different clinical profile and worse outcomes, which makes it imperative to investigate the pattern of involvement of known modifiable and non-modifiable risk factors in local contexts. This data is useful for informing policy and directing health system planning especially as it regards to women’s health. Data on BP trajectory as well as sex dissimilarities in the trajectory of CV risk factors in sub-Saharan Africa is limited [25–29].

We thus set out to conduct a sex-based comparison of the correlation and association between age and blood pressures as well as specific anthropometric indices (waist circumference and BMI) from early adulthood.

## Methods

We conducted a secondary analysis of the data from a cross-sectional, community-based hypertension prevalence study in Nigerians aged 16 years and above, residing in an urban area of Lagos state, Nigeria and whose methodology has previously been described [30]. To summarize, between May and December 2017 after obtaining ethics approval from the Lagos University Teaching Hospital Health Research and Ethics Committee, we carried out a cross-sectional prevalence study of blood pressure profiles: using a stratified multistage random sampling approach we carried out a door-to-door survey of 200 households randomly selected from 8 of 16 mixed income densely populated local government areas of the state [30]. As previously described, we utilized the World Health Organizations STEPwise approach to chronic disease risk factor surveillance (WHO STEPS) [30, 31]. Written informed consent was also obtained from the head of household and/or legal guardian, and from each participating individual. The study protocol was carried out in accordance with the Declaration of Helsinki.

Blood pressure was measured using an appropriately calibrated Omron® sphygmomanometer, with the average of the last two (of three) readings taken while seated utilized for the study [30]. Anthropometric indices i.e., weight, height*,* waist circumference, hip circumference and waist–hip ratio were all measured according to standard protocol and this has been previously documented [32–34] Data for 5135 community-dwelling participants were included in this secondary analysis and the variables of interest include age (years), sex, systolic BP (mmHg), diastolic BP (mmHg), BMI (kg/m^2^) and waist circumference (cm). Pulse pressure (mmHg) was derived as the difference between systolic BP and diastolic BP (systolic BP minus diastolic BP).

### Data analysis

Data was analyzed using IBM ® SPSS ® version 24. Continuous variables are presented as mean ± standard deviation (SD) and inter-group comparisons were explored using Analysis of Variance (ANOVA). Correlation analysis (Spearman’s) was conducted to explore the association between age and systolic blood pressure (SBP), diastolic blood pressure (DBP), pulse pressure (PP), body mass index (BMI), and waist circumference (WC) in males compared with females. Based on the age at which the male and female SBP and PP intercepted each other (45 years), the mean difference in variables between those aged < and ≥ 45 years was computed using Medcalc® open-source software, and presented as mean difference (95% confidence interval) [35].

Generalized linear regression was used to analyze the influence of age (as age categories) and sex (female as reference category) as factors on SBP, DBP, PP (with BMI and WC added as co-variates). This was carried out as individual main effects as well as interaction term (age category*sex). Generalized linear regression was also used to analyze the influence of age (as age categories) and sex (female as reference category) on WC and BMI (also analyzing main effects and interaction of age category*sex). The regression models out-performed the null hypothesis as the omnibus test was significant for all (*p* = 0.000) and the models were all a good fit to the data as deviance (goodness-of-fit test) was < 2.5 for all models (0.02–1.58). Statistical significance was set at the level of *p* < 0.05.

## Results

**Fig. 1:**
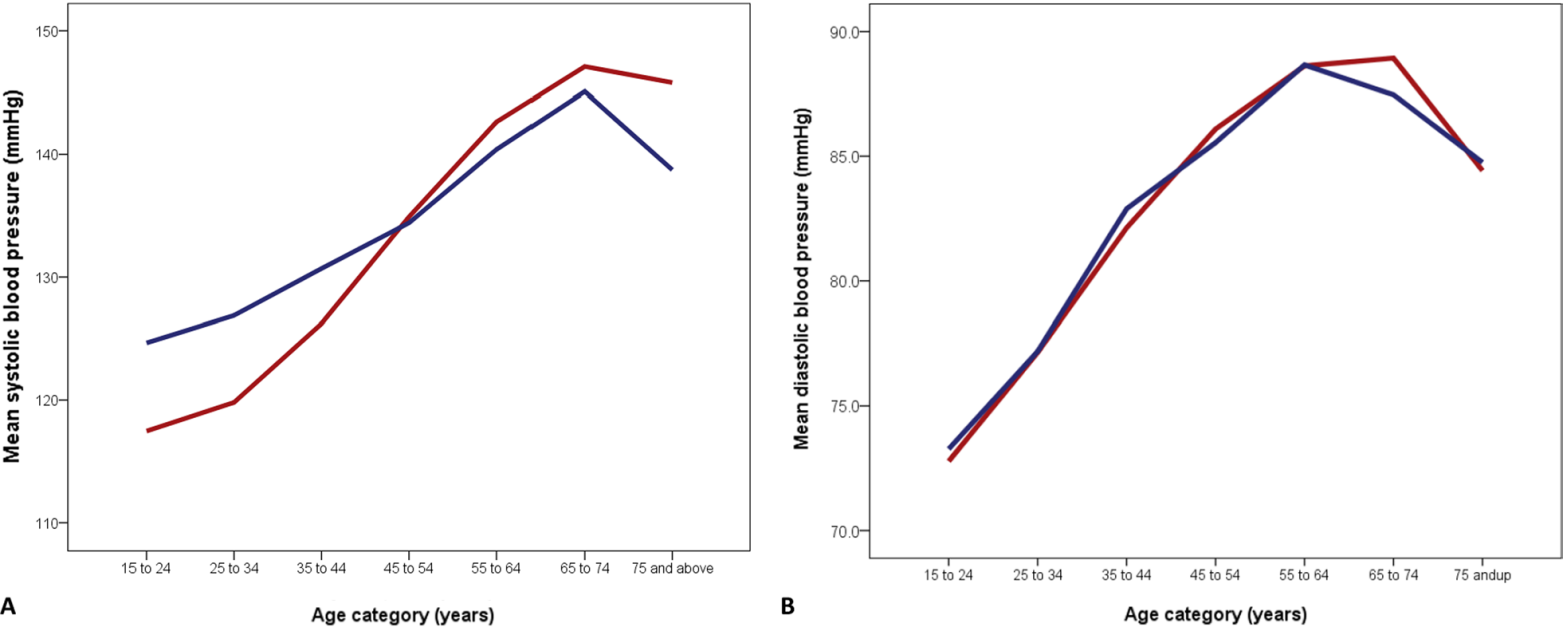
Trajectory of association between mean blood pressures and age based on sex. Line graphs depicting the trend of association between mean blood pressures and age in females (red) and males (blue): **A** – systolic blood pressure; **B** – diastolic blood pressure

There were 5135 urban community-dwelling adults (age range 16 to 92 years) comprising 2671 (52.0%) females and 2464 (48.0%) males. Table 1 shows the characteristics of the study population, including a comparison of blood pressure and anthropometric indices. We dichotomized age at 45 years based on the observation that the sex-based intercept for SBP trajectory with age was in that age stratum, (Fig. 1). There was no sex difference in the mean ages of the population, nor of the proportion of adults < or ≥ 45 years based on gender (*p* = 0.24). Males had significantly higher SBP and PP compared to females only below 45 years (*p* < 0.001). All the mean BP measures (SBP, DBP, and PP) were higher in women aged ≥ 45 years although the difference did not reach statistical significance. Diastolic blood pressure was comparable irrespective of sex in the age strata. BMI and WC were significantly higher in females overall and in the age categories < 45 and ≥ 45 years (*p* < 0.001). The mean differences in the measured variables between age categories < 45 and ≥ 45 years in both sexes are also shown in Table 1, and indicate that females compared to males, had consistently greater positive differences from age categories < 45 to ≥ 45 years for all the variables.

**Table 1:** Characteristics of study participants (overall and dichotomized by age below and above 45 years)

Variable	Female n = 2671	Male n = 2464	*p* value
Mean age ± SD, *years*	37.4 ± 13.3	37.7 ± 12.8	0.36
Age distribution, *n (%)* < 45 years ≥ 45 years	1995 (74.7) 676 (25.3)	1818 (73.8) 646 (26.2)	0.24
Mean systolic BP ± SD, mmHg All ages < 45 years ≥ 45 years	124.7 ± 19.6 120.3 ± 16.2 137.7 ± 22.6	128.9 ± 17.3 126.4 ± 15.1 135.6 ± 20.9	< 0.001 < 0.001 0.09
Mean difference (95% CI) in SBP (male v. female) for < 45 years	6.1(5.1 to 7.10)^a^		< 0.0001
Mean difference (95% CI) in SBP (male v. female) for ≥ 45 years	−2.1 (−4.45 to 0.25)^a^		0.08
Mean difference (95% CI) in SBP (< 45 v. ≥ 45 years)	17.4 (15.8 to 19.0) *p* < 0.0001	9.2 (7.7 to 10.7) *P* < 0.0001	
Mean diastolic BP ± SD, mmHg All ages < 45 years ≥ 45 years	80.4 ± 13.3 78.1 ± 12.4 87.1 ± 13.7	80.8 ± 13.2 78.7 ± 12.3 86.5 ± 13.8	0.29 0.12 0.44
Mean difference (95% CI) in DBP (male v. female) for < 45 years	0.60 (0.19 to 1.38)^a^		0.13
Mean difference (95% CI) in DBP (male v. female) for ≥ 45 years	−0.60 (−2.08 to 0.88)^a^		0.43
Mean difference (95% CI) in DBP (< 45 v. ≥ 45 years)	9.0 (7.9 to 10.1) *P* < 0.0001	7.8 (6.7 to 8.9) *P* < 0.0001	
Mean pulse pressure ± SD, mmHg All ages < 45 years ≥ 45 years	44.3 ± 12.9 42.2 ± 11.2 50.6 ± 15.5	48.1 ± 12.2 47.7 ± 11.5 49.1 ± 14.1	< 0.001 < 0.001 0.08
Mean difference (95% CI) in PP (male v. female) for < 45 years	5.50 (4.78 to 6.22)^a^		< 0.0001
Mean difference (95% CI) in PP (male v. female) for ≥ 45 years	−1.50 (−3.10 to 0.10)^a^		0.07
Mean difference (95% CI) in PP (< 45 v. ≥ 45 years)	8.4 (7.3 to 9.5) *P* < 0.0001	1.4 (0.3 to 2.5) *P* = 0.013	
Mean waist circumference ± SD, cm All ages < 45 years ≥ 45 years	89.6 ± 15.0 87.6 ± 14.4 95.8 ± 14.9	84.4 ± 11.6 82.9 ± 11.1 88.6 ± 11.9	< 0.001 < 0.001 < 0.001
Mean difference (95% CI) in WC (male v. female) for < 45 years	−4.70 (−5.52 to −3.88)^a^		< 0.0001
Mean difference (95% CI) in WC (male v. female) for ≥ 45 years	−7.20 (−8.66 to −5.74)^a^		< 0.0001
Mean difference (95% CI) in WC (< 45 v. ≥ 45 years)	8.2 (6.9 to 9.5) *P* < 0.0001	5.7 (4.7 to 6.7) *P* < 0.0001	
Mean body mass index ± SD, kg/m^2^ All ages < 45 years ≥ 45 years	25.0 ± 5.8 24.5 ± 5.5 26.7 ± 6.2	24.2 ± 4.9 23.9 ± 4.7 25.1 ± 5.5	< 0.001 < 0.001 < 0.001
Mean difference (95% CI) in BMI (male v. female) for < 45 years	−0.60 (−0.93 to −0.27)^a^		0.0003
Mean difference (95% CI) in BMI (male v. female) for ≥ 45 years	−1.60 (−2.23 to −0.97)^a^		< 0.0001
Mean difference (95% CI) in BMI (< 45 v. ≥ 45 years)	2.2 (1.7 to 2.7) *P* < 0.0001	1.2 (0.8 to 1.6) *P* < 0.0001	

*PP* pulse pressure; *DBP* diastolic blood pressure; *SBP* systolic blood pressure; *BMI* body mass index; *WC* waist circumference; *SD* standard deviation; *CI* confidence interval

^a^Direction of mean difference: positive = higher male value; negative = higher female value

The blood pressures and anthropometric indices trajectories with age in males and females are illustrated in Table 2 and Fig. 1. As shown in Table 3, in correlation analysis, all SBP, DBP, BMI and WC exhibited significant positive correlation with age in both sexes (*p* < 0.001), whereas PP correlated significantly with age only in females. There was a stronger correlation with age for all the parameters in females compared to males.

**Table 2:** Comparison of blood pressures and anthropometric indices by mid-decade age category and sex

Age category (years)	Gender (n)	Systolic BP (mmHg)		Diastolic BP (mmHg)		Pulse pressure (mmHg)		Body mass index (kg/m^2^)		Waist circumference (cm)	
			*p*		*p*		*P*		*p*		*p*
15–24	F (477)	115.9 ± 12.3	0.000	72.8 ± 9.6	0.46	43.1 ± 10.7	0.000	22.4 ± 5.2	0.81*	80.7 ± 12.2	0.07*
	M (416)	123.0 ± 12.3		73.3 ± 10.4		49.7 ± 10.2		22.3 ± 4.1		79.3 ± 9.7	
25–34	F (722)	118.4 ± 15.2	0.000*	77.1 ± 11.7	0.91*	41.2 ± 10.2	0.000*	24.1 ± 5.2	0.004*	87.2 ± 13.3	0.000*
	M (630)	125.1 ± 13.2		77.2 ± 10.4		47.9 ± 11.2		23.4 ± 4.4		82.0 ± 9.8	
35–44	F (796)	124.8 ± 18.0	0.000	82.1 ± 13.1	0.25	42.6 ± 12.1	0.000*	26.1 ± 5.5	0.000*	92.1 ± 14.9	0.000*
	M (772)	129.4 ± 17.3		82.9 ± 13.2		46.5 ± 12.2		25.1 ± 4.9		85.6 ± 12.1	
45–54	F (368)	133.2 ± 20.8	0.88	86.1 ± 13.1	0.58	47.1 ± 13.6	0.74	27.1 ± 6.1	0.002*	95.2 ± 14.6	0.000*
	M (387)	133.0 ± 20.0		85.5 ± 13.8		47.4 ± 12.9		25.8 ± 5.8		88.2 ± 11.5	
55–64	F (190)	141.7 ± 24.5	0.23*	88.6 ± 14.8	0.98	53.0 ± 16.5	0.08	26.6 ± 6.3	0.001*	97.3 ± 15.4	0.000*
	M (171)	138.7 ± 22.0		88.7 ± 14.2		50.1 ± 14.8		24.6 ± 4.8		89.1 ± 13.0	
65–74	F (86)	145.3 ± 20.4	0.61	88.9 ± 12.6	0.52	56.3 ± 15.9	0.90	26.3 ± 5.1	0.000	96.9 ± 13.5	0.007
	M (58)	143.4 ± 22.2		87.5 ± 14.4		56.0 ± 17.9		23.4 ± 4.0		91.0 ± 11.8	
≥ 75	F (32)	145.3 ± 25.9	0.18	84.4 ± 15.2	0.90*	60.9 ± 17.0	0.04	24.1 ± 8.6	0.73	91.1 ± 17.9	0.19
	M (30)	137.5 ± 17.5		84.8 ± 9.1		52.8 ± 12.8		23.5 ± 6.7		86.1 ± 11.3	

*F *Female; *M* Male; *BP* Blood Pressure. All values are mean ± standard deviation (SD)

^*^Welch Brown Forsythe statistic reported as Levene’s significant and homogeneity of variances could not be assumed

**Table 3:** Spearman correlation analysis (r_s_) of age compared to blood pressures and anthropometric indices

Variable	Female n = 2671 r_s_ (*p* value)	Male n = 2464 r_s_ (*p* value)
Systolic blood pressure, mmHg	0.422 (< 0.001)	0.264 (< 0.001)
Diastolic blood pressure, mmHg	0.402 (< 0.001)	0.389 (< 0.001)
Pulse pressure, mmHg	0.205 (< 0.001)	−0.03 (0.13)^a^
Body mass index, kg/m^2^	0.309 (< 0.001)	0.237 (< 0.001)
Waist circumference, centimeters	0.383 (< 0.001)	0.310 (< 0.001)

All comparisons are between age and the stated variable. r_s_ Spearman rank correlation coefficient

^a^Not statistically significant

### Trajectory of blood pressure with age based on sex

Table 2 and Fig. 1 show the trends in mean SBP and DBP by age category for both sexes. From the age group 25 to 34 years, SBP demonstrated a steeper rise in in females, intercepting and becoming higher than in men from the age category 45 to 54 years upwards. From age 65 years there was a decline in the SBP, albeit more obvious in males. The upward trajectory in DBP was parallel in both sexes, with nearly overlapping mean values (*p* < 0.05). Pulse pressure had a similar course with SBP (Table 2).

### Trajectory of BMI and WC with age group based on sex

**Fig. 2:**
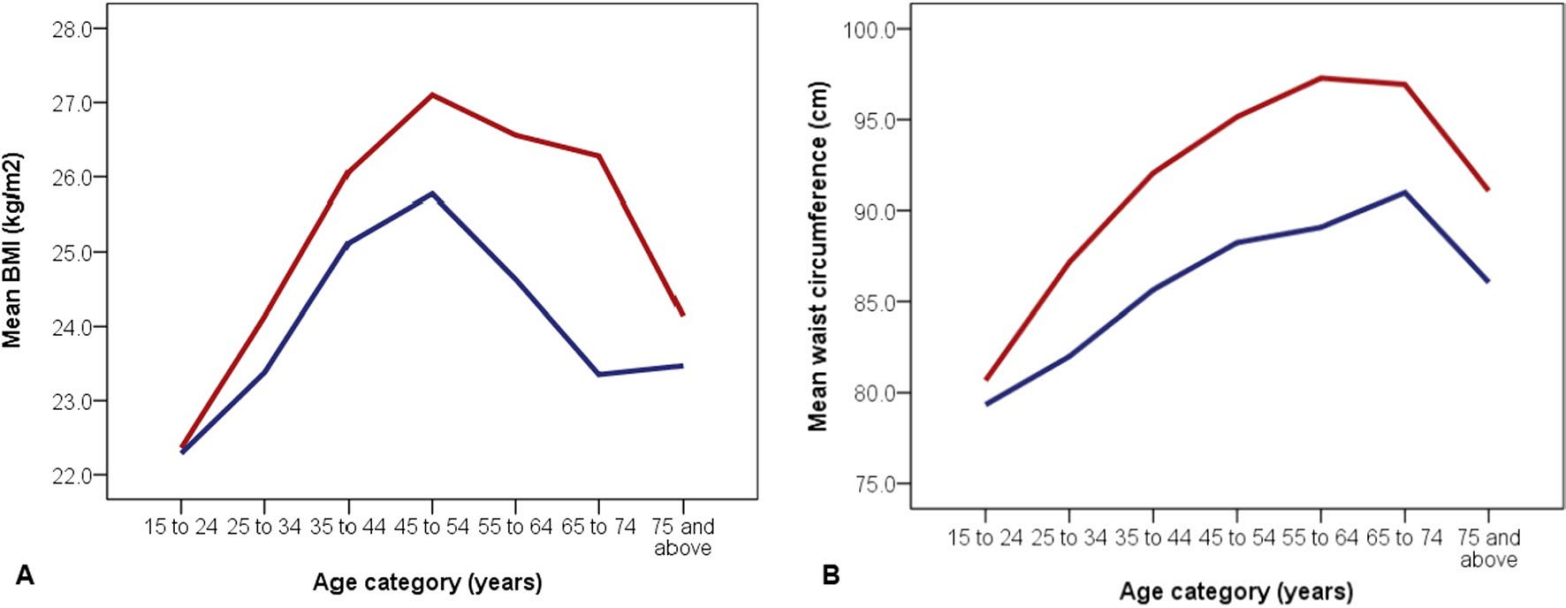
Trajectory of association between anthropometric indices (BMI and WC) and age based on sex. Line graphs depicting the trend of association between anthropometric indices and age in females (red) and males (blue): **A** – BMI; **B** – waist circumference (WC). Both BMI and WC were higher in females compared to males across all age categories, with significant differences across all age groups except ≤ 24 and ≥ 75 (*p* > 0.05)

The trend of association between anthropometric indices and age in males and females are displayed in Table 2 and Fig. 2. In both sexes there was a steady rise in anthropometric indices with age, with decline in mean BMI and waist circumference observed from age categories 45 to 54 years and 65 to 74 years respectively. The decline in BMI with age was steeper in males than in females.

### Predictors of BP and anthropometric profiles and their interactive effects

Generalized linear regression results are displayed in Additional file 1: Table 1. For models involving BP profiles (SBP, DBP and PP), the ≥ 75-year category was redundant. All variables in the model had significant Type III effects (sex, age category, BMI and WC). The interaction model (interaction term age category* sex) revealed that SBP was significantly predicted by age category in females from ages 15–54 years while DBP was only significantly predicted by age category in females in the 15–34-year range (*p* < 0.01). BMI and WC were significantly predicted by age category in females, only in the 25–34-year category (*p* < 0.01). (Additional file 1: Table 1).

## Discussion

The impetus for this study was the reported sex disparity in attributable risk for adverse cardiovascular outcomes (with a higher burden in females) from other populations. Our objective was to gain insight into the trajectories of blood pressure and anthropometric indices with increasing age based on gender in our black African population.

With respect to blood pressures, we observed that, compared to males, SBP was initially lower in females at adolescence, but rose steeply from age group 25–34, intersected with and became higher than males at 45–54 years, only declining in both sexes from age bracket 65–74 years (but with a more appreciable decline in men). Diastolic BP had similar course in both sexes up to age 55–64 group, when it subsequently declined in men but continued to rise in women till age 65–74 where a steep decline was observed to parallel the values for men by age ≥ 75 years. The rise in pulse pressure also occurred at an earlier age in females (25–34 v. 35–44), intersected at 45–54 years, continuing to rise in females while declining in men from age ≥ 65 years.

Our findings corroborate those from other populations indicating that SBP and DBP are lower in females in early life, subsequently exhibiting a steeper and more rapid rise in SBP with advancing age, switching to become higher at about middle age, whereas the trajectory of DBP remains largely similar in both sexes [18, 36] This pattern suggests a greater cardiovascular risk in women from middle age, coinciding with the menopausal age in many populations [1, 7, 8, 18, 19, 37, 38]. The consequent loss of the protective effect of oestrogen at menopause with resultant increased renal sodium retention, loss of endothelial dependent nitric oxide production, increase in plasma rennin and angiotensin converting enzyme activity may partially account for the observation [18]. However, we postulate that the higher risk actually precedes menopause based on our observation (and that of others), that women actually begin to exhibit a sharper and consistent rise in SBP from about the age of 25 years (at least 2 decades before typical menopausal age), and at a rate steeper than that observed from middle age [36]. This observation is important as it is well documented that each 20 mmHg increase in SBP is associated with a doubling in the risk of adverse CV events [39]. It would therefore appear that our population of black African women actually bear a greater CV risk at a much earlier age, and is consistent with previous reports that sub-Saharan Africans have a lower age of onset and different clinical profile of cardiovascular diseases [28, 29]. Existing estimates of CVD risk from most sub-Saharan African countries are premised on sparse, sometimes methodologically flawed primary studies, and probably represent an underestimation of the true burden in both sexes, with the potential for greater misrepresentation of females. This would invariably misdirect surveillance and the timing of gender specific interventions if the surge in SBP observed earlier in life in females is not taken into consideration. More so, as previously reported, the steady increase in SBP (hence the CV risk) is sustained throughout life in females [36]. However, in the European cohorts aged 19–78 years included in the MORGAM (MOnica, Risk, Genetics, Archiving, and Monograph) project, the relative superiority of SBP over DBP as a risk factor for fatal and non-fatal stroke risk which was consistent and independent of other cardiovascular risk factors, became apparent at age 47 years, and was significant from age 62 years [40]. The study also demonstrated a significant effect modification of the SBP-stroke mortality association by sex in which the positive association between SBP and stroke mortality was present in both sexes but did not reach significance in women before middle age (implying a pre-menopausal hormonal protection up to age 50 or around menopause) in that population [40, 41].

The pulse pressure trajectory reported in our study is similar to that for SBP and is in keeping with previous studies [8, 36]. Pulse pressure is independently associated with subclinical cardiovascular diseases, albeit with sex variability (increased tendency in females) in the strength of this association [8, 36]. For example, increased pulse pressure is more strongly associated with increased left ventricular mass index, increased prevalence of, and poorer outcome from heart failure with preserved ejection fraction in females [8, 36, 42].

The GBD Study 2019 reiterates the disturbing burden of metabolic risk factors including BMI as leading causes of DALYs and contributors to mortality globally, with considerable heterogeneity in the risks and trends between countries [1]. High BMI is one of three risk factors that accounted for > 1% DALYs in addition to increasing in exposure by more than 1% per annum [1]. The higher BMI and WC in black African females in this study, consistent across all age groups, corroborate reports from several other studies [43–45]. However, it differs from some studies conducted amongst predominantly Caucasian and Asian populations where men had higher waist circumference, and in keeping with recognized ethnic and regional variability in sex distribution of abdominal adiposity [43–48]. Black women reportedly have a greater shift in waist circumference with age and an increased tendency to truncal obesity compared to other ethnicities and men [45]. Both increasing BMI and WC are independently associated with increased risk of hypertension and cardiovascular diseases and may contribute to the steady rise in blood pressure with age observed in this and other studies [21, 22, 49–51]. Sexual dimorphism in the pathogenesis of obesity-related hypertension has been associated with increased atherogenic lipid profile and induction of metabolic disorders in women but not in men [52]. This may further widen the gender divide culminating in the tendency towards a worse CV risk profile and potential for worse CV outcomes in women, even starting at an earlier age.

We acknowledge the limitations of our study in being largely descriptive and cross-sectional data for which only one set of measures (blood pressure and anthropometry) were obtained, and did not include any longitudinal outcome data to test the impact of our observation. In deriving conclusions regarding the implications of our findings, we have relied on existing literature that indicate the strength of association between high systolic blood pressure and anthropometric indices (e.g. BMI) and adverse CV outcome, such as that from the GBD 2019 study. The smaller numbers of participants at the highest extremes of age in this study (≥ 75 years) may also have introduced some bias into the data in that subgroup for both sexes. The numbers in this study represent the small proportion of the general population in the country (2.74%) aged ≥ 65 years according to estimates from the United Nations Population Divisions’ World Population prospects (2019 revision) [53]. Otherwise, the robust sample size and the population source of our data with adequate representation of other age strata may however attenuate this limitation, although we suggest longitudinally studying a larger and nationally representative sample of the population as well as the older elderly in the future, given improvements in life expectancy that are expanding the population representation of that age group. Our primary data collection did not include laboratory-based assessments and, although we appreciate the import of exploring other metabolic variables that can further substantiate cardiovascular risk; this consideration was precluded by funding and logistic limitations.

## Conclusions

The trajectory of SBP, PP, and anthropometric indices of obesity and abdominal adiposity documented in our study, and taken together with existing literature are persuasive indicators of the need to focus on women’s cardiovascular risk modification, starting in early adolescence through to the postmenopausal period. We join previous researchers to advocate for sex specific intervention strategies to modify the trend [3–5, 12–14]. In black African females in our population, we propose population-level primordial prevention policies and projects aimed at increasing awareness on the specific risk of women, improving access to information regarding healthy lifestyle choices from early life, and enabling implementation of evidence-based measures such as dietary choices and exercise. In addition, primary prevention through policies and programs that improve access to documenting and tracking women’s cardiovascular health indices and outcomes across the lifespan, integrated into existing women’s health or female-predominant user programs (such as maternity and childhood immunization) would be beneficial.

## Supplementary Information

**Additional file 1: Table 1.** Generalized linear regression models using age and sex to predict trend in blood pressure profiles and anthropometric indices.

## Data Availability

The datasets used and/or analyzed during the current study are available from the corresponding author on reasonable request.

## Abbreviations

ANOVA: Analysis of variance
BP: Blood pressure
BMI: Body mass index
CV: Cardiovascular
CVD: Cardiovascular disease
DBP: Diastolic blood pressure
DALYs: Disability-adjusted life years
GBD: Global burden of diseases
IBM: International business machine corporations
LUTH-HREC: Ethics approval was obtained from the Lagos university teaching hospital health research ethics committee.
PP: Pulse pressure
SBP: Systolic blood pressure
SD: Standard deviation
SPSS: Statistical package for social sciences
SSA: Sub-Saharan Africa
WC: Waist circumference
WHO STEPS: World Health Organizations STEPwise approach to chronic disease risk factor surveillance
