# Supplementary material for: The impact of sex on blood pressure and anthropometry trajectories from early adulthood in a Nigerian population: insights into women’s cardiovascular disease risk across the lifespan
Source: BMC Womens Health. 2022 Jul 22;22:303. doi: 10.1186/s12905-022-01888-7 (PMC9306031; doi:10.1186/s12905-022-01888-7)
Supplement: Supplementary file 1 — Additional file 1: Table 1. Generalized linear regression models using age and sex to predict trend in blood pressure profiles and anthropometric indices. [file 12905_2022_1888_MOESM1_ESM.docx]

**Additional file 1: Table 1: Generalized linear regression models using age and sex to predict trend in blood pressure profiles and anthropometric indices.**

| **Variables** | **Standard error** | **Wald Chi-square** | **df** | **p** | **95% C.I.** | |
| --- | --- | --- | --- | --- | --- | --- |
|  |  |  |  |  | **Lower** | **Upper** |
| **Systolic blood pressure*** | | | | | | |
| **Intercept** | 0.0218 | 46375.530 | 1 | <0.0001 | 4.660 | 4.746 |
| **Sex = Female** | 0.0036 | 138.235 | 1 | <0.0001 | 0.036 | 0.050 |
| **Age category** |  |  |  |  |  |  |
| **15 – 24 years** | 0.0168 | 88.104 | 1 | <0.0001 | -0.190 | -0.125 |
| **25 – 34 years** | 0.0166 | 80.810 | 1 | <0.0001 | -0.181 | -0.116 |
| **35 – 44 years** | 0.0165 | 50.198 | 1 | <0.0001 | -0.149 | -0.085 |
| **45 – 54 years** | 0.0169 | 20.851 | 1 | <0.0001 | -0.110 | -0.044 |
| **55 – 64 years** | 0.0175 | 1.499 | 1 | 0.221 | -0.056 | 0.013 |
| **65 – 74 years** | 0.0194 | 0.431 | 1 | 0.512 | -0.025 | 0.051 |
| **BMI (kg/m^2^)** | 0.0004 | 87.551 | 1 | <0.0001 | 0.003 | 0.004 |
| **Waist circumference (cm)** | 0.0002 | 49.228 | 1 | <0.0001 | 0.001 | 0.001 |
| **Diastolic blood pressure*** | | | | | | |
| **Intercept** | 0.0250 | 28826.316 | 1 | <0.0001 | 4.197 | 4.295 |
| **Sex = Female** | 0.0042 | 7.985 | 1 | 0.005 | 0.004 | 0.020 |
| **Age category** |  |  |  |  |  |  |
| **15 – 24 years** | 0.0192 | 47.990 | 1 | <0.0001 | -0.170 | -0.095 |
| **25 – 34 years** | 0.0189 | 21.399 | 1 | <0.0001 | -0.125 | -0.050 |
| **35 – 44 years** | 0.0189 | 2.866 | 1 | 0.090 | -0.069 | 0.005 |
| **45 – 54 years** | 0.0193 | 0.009 | 1 | 0.924 | -0.036 | 0.040 |
| **55 – 64 years** | 0.0200 | 3.254 | 1 | 0.071 | -0.003 | 0.075 |
| **65 – 74 years** | 0.0221 | 2.386 | 1 | 0.122 | -0.009 | 0.078 |
| **BMI (kg/m^2^)** | 0.0005 | 64.137 | 1 | <0.0001 | 0.003 | 0.004 |
| **Waist circumference** | 0.0002 | 28.818 | 1 | <0.0001 | 0.001 | 0.001 |
| **Pulse Pressure*** | | | | | | |
| **Intercept** | 0.0441 | 7027.413 | 1 | <0.0001 | 3.614 | 3.787 |
| **Sex = Female** | 0.0074 | 168.148 | 1 | <0.0001 | 0.081 | 0.110 |
| **Age category** |  |  |  |  |  |  |
| **15 – 24 years** | 0.0341 | 33.736 | 1 | <0.0001 | -0.265 | -0.131 |
| **25 – 34 years** | 0.0336 | 55.940 | 1 | <0.0001 | -0.317 | -0.186 |
| **35 – 44 years** | 0.0335 | 60.152 | 1 | <0.0001 | -0.326 | -0.194 |
| **45 – 54 years** | 0.0342 | 36.950 | 1 | <0.0001 | -0.275 | -0.141 |
| **55 – 64 years** | 0.0356 | 10.301 | 1 | 0.001 | -0.184 | -0.044 |
| **65 – 74 years** | 0.0394 | 0.117 | 1 | 0.732 | -0.091 | 0.064 |
| **BMI (kg/m^2^)** | 0.0008 | 21.838 | 1 | <0.0001 | 0.002 | 0.005 |
| **Waist circumference (cm)** | 0.0003 | 16.680 | 1 | <0.0001 | 0.001 | 0.002 |
| **Body Mass Index^#^** | | | | | | |
| **Threshold (BMI class):**  **Normal**  **Overweight**  **Obese** | 0.3332  0.3366  0.3344 | 11.598  113.894  26.939 | 1  1  1 | 0.001  <0.0001  <0.0001 | 0.482  2.932  1.080 | 1.788  4.252  2.391 |
| **Sex = Female** | 0.0555 | 24.696 | 1 | <0.0001 | -0.385 | -0.167 |
| **Age category** |  |  |  |  |  |  |
| **15 – 24 years** | 0.2609 | 2.336 | 1 | 0.126 | -0.910 | 0.113 |
| **25 – 34 years** | 0.2565 | 5.574 | 1 | 0.018 | -1.109 | -0.103 |
| **35 – 44 years** | 0.2546 | 0.952 | 1 | 0.329 | -0.748 | 0.251 |
| **45 – 54 years** | 0.2587 | 0.628 | 1 | 0.428 | -0.712 | 0.302 |
| **55 – 64 years** | 0.2682 | 0.513 | 1 | 0.474 | -0.718 | 0.334 |
| **65 – 74 years** | 0.2948 | 2.857 | 1 | 0.091 | -1.076 | 0.079 |
| **Waist circumference (cm)** | 0.0020 | 99.401 | 1 | <0.0001 | 0.016 | 0.024 |
| **Waist circumference Category (IDF)^&^** | | | | | | |
| **Intercept** | 0.3382 | 55.475 | 1 | <0.0001 | 1.856 | 3.182 |
| **Sex = Female** | 0.0804 | 1086.872 | 1 | <0.0001 | -2.807 | -2.492 |
| **Age category** |  |  |  |  |  |  |
| **15 – 24 years** | 0.3317 | 18.602 | 1 | <0.0001 | -2.081 | -0.780 |
| **25 – 34 years** | 0.3263 | 5.282 | 1 | 0.022 | -1.389 | -0.110 |
| **35 – 44 years** | 0.3256 | 1.179 | 1 | 0.278 | -0.992 | 0.285 |
| **45 – 54 years** | 0.3331 | 0.112 | 1 | 0.738 | -0.765 | 0.541 |
| **55 – 64 years** | 0.3476 | 0.401 | 1 | 0.527 | -0.461 | 0.901 |
| **65 – 74 years** | 0.3960 | 3.587 | 1 | 0.058 | -0.026 | 1.526 |
| **BMI (Kg/m^2^)** | 0.0518 | 552.730 | 1 | <0.0001 | 1.117 | 1.320 |

Footnote: BMI = body mass index; BP = Blood pressure; CI = confidence interval; IDF = International Diabetes Federation. All models are generalized linear models with link identity function. Models were as follows: *gamma with loglink, ^#^ - ordinal logistic and ^&^ - binary logistic. Results displayed are for generalized regression models using interaction terms age category*sex, in addition to the factors and co-variates described in text (results).
